# Postfeminist Healthism: Understanding the Gendering of Healthism Using Menstrual Tracking Apps as an Example

**DOI:** 10.1111/1467-9566.70116

**Published:** 2025-11-16

**Authors:** Sarah Riley, Adrienne Evans, Martine Robson

**Affiliations:** ^1^ School of Psychology Massey University Wellington New Zealand; ^2^ Gender and Culture Coventry University Coventry UK; ^3^ Department of Psychology Aberystwyth University Aberystwyth UK

**Keywords:** gender, healthism, menstrual tracking apps, postfeminist healthism, postfeminist sensibility

## Abstract

‘Postfeminist healthism’ offers an essential framework for understanding how healthism is gendered. In this article, we describe and advance the concept of postfeminist sensibility and its synergistic alignments with healthism. We then consider how postfeminist healthism operates as a subjectifying force for the millions of girls, women, and other feminine‐identified people globally—even when it harms their mental or physical health. We use menstrual tracking apps (MTAs) as an indicative example to both demonstrate how a postfeminist healthism acts at the intersections of bodies, subjectivity, and health, and to show the value of a postfeminist healthism in understanding MTAs. Overall, we show the importance of understanding the distinct ways in which healthism is gendered through postfeminism.

## 1 Introduction

Gender is not central to discussions of healthism, despite obvious links between gender, health, and healthism. For example, women are more likely to be diagnosed later than men across 700 diseases (Westergaard et al. 2019) and face stark inequalities in health related to classed, racialised, socio‐economic and disability status (Allen and Sesti 2018). Paradoxically, women's out of pocket spending on healthcare is higher—in the United Kingdom for example, it is 50% higher than men's across all categories including diagnostics and wearables, reproductive and general health, and counselling, a £1.5 billion difference (Hampson and King 2024). Women are also more responsibilised for their own and other's health, including their male partner if they have one (Riley et al. 2019). It is therefore important to understand how contemporary gendered discourses and their subjective effects intersect with health and healthism. In this article we propose ‘postfeminist healthism’ as offering such an interpretive framework.

To develop our argument, we first outline healthism and the limited scholarship exploring connections between healthism and gender, demonstrating that heathism is always already gendered, but this relationship is under‐theorised. We then show how a postfeminist sensibility is an important way to understand this form of gendering, because postfeminist sensibility is both a hegemonic framing of gender and imbricated with healthism. By tracing iterations of a postfeminist sensibility and its entanglements with health, we advance existing conceptualisations of postfeminist healthism. We draw together a range of arguments dispersed across the literature and, building on these, offer a novel analysis of how postfeminist healthism operates as a powerful subjective force—even in the face of critique or recognisable harm.

To illustrate these arguments, and contribute to the field of digital health, we thread an analysis of menstruation tracking apps (MTAs) throughout, drawing on a combination of our own data,^1^ material from apps and online tools, and examples from existing MTA literature. MTAs are an important site to examine the gendering of healthism as they are a technology designed specifically with women's health needs in mind that often map onto a healthism framework. MTAs are digital apps that provide users with tools to track their menstrual cycle. Part of much larger ecologies of digital self‐tracking technology for health, MTAs are normalised in the management and monitoring of menstruation by many menstruators (current estimates at 500 million, Rampazzo et al. 2024) as well as the healthcare providers and professionals who advise them.^2^ MTAs are a significant part of the enormously profitable sector for digital health technology, with thousands of apps available.

MTAs have become a growth area in academic research into digital health (Riley et al. 2025), but are rarely considered through the lens of a postfeminist healthism. This paucity relates to how discussions of a postfeminist sensibility often pre‐date, or have limited engagement with, the digital technologies that shape today's health landscape. Concurrently, although healthism is a key term in analysing self‐tracking technology (see e.g., Kent 2023; Lupton 2013, 2014), few have used postfeminism to make sense of this technology, despite a highly femininised address that evokes discourses of empowerment, yet disciplines and regulates its users—key tropes of postfeminism (e.g., Tylstedt et al. 2023; Ford et al. 2021; Hendl and Jansky 2022; see Polzer et al. 2022; Riley and Paskova 2022 for exceptions). In analysing MTAs through the lens of postfeminist healthism, we therefore provide new insights for health research, where health is always already gendered, as well as apply postfeminist healthism to MTA where it could be highly relevant for developing the field. Overall, our contribution is in advancing the field of healthism and social science research of menstruation tracking apps through explicating the concept of postfeminist healthism.

### 1.1 Healthism and Gender

‘Healthism’ describes a cultural shift towards an expectation to achieve a stable state of health, enabled through lifestyle choices and consumer practices (Crawford 1980, 2006). This orientation produces ‘health’ as a highly valued characteristic of a person, enacted through self‐mastery, self‐discipline, and engagement with health and wellness practices. As such, health is individualised, and individuals are ‘responsiblised’ for attaining health and well‐being, something that is well‐recognised in the digital health literature (e.g., Kent 2023; Lupton 2013). For example, users of one of the most popular MTAs, ‘Flo’, must agree that they ‘will use Flo to always know what's coming, manage how I feel, and enhance my life’.^3^ Situated within the ‘health and wellness’ part of app purchasing platforms, Flo offers a commercialised health practice—with premium subscriptions for different services—that constructs expectations for individuals to use this technology to meet valued understandings of embodiment, namely, to predict, manage, and optimise their life. Similarly, Natural Cycles states on their website that their goal is ‘empowering every woman with the knowledge that she needs to take charge of her health’. MTAs are unlike most tracking apps because they involve tracking bodily processes that are out of the control of the person, but even in this context, a pattern emerges in which menstrual health is a choice to know, take charge of, and own is evident.

Healthism contrasts with thinking of health defined in terms of illness and sickness, or in relation to governmental provision of public health services. Instead, connecting with neoliberal discourses of risk, healthism constructs health as expected, but always at risk (Lupton 2013). This risk can be a successfully managed by the disciplined, knowledgeable individual—supported by experts (including MTAs). However, health unknowns and the inevitability of ill health and death mean that the notion of control is an illusion. In Crawford's analysis of healthism, we therefore see a form of ‘cruel optimism’ (Berlant 2011), where people's desires are attached towards a particular object (i.e., ‘good’ health) that is ultimately toxic or unattainable. Although menstrual cycles cannot be controlled, the discourse of risk management still applies—for example, expectations that MTA use will enable conception (managing the risk of infertility), or allow users to learn to manage their responses to their cycles (managing risks related to poor mental health or low productivity). When this works, people can experience a sense of empowerment, but the risk of cruel optimism remains, for example when pregnancies do not materialise (Hamper 2020).

Healthism addresses everyone to be always working on achieving health, when health covers many, if not all, aspects of life. Thus, people can ‘talk about having a healthy lifestyle, a healthy relationship, a healthy work–life balance or a healthy positive attitude’ (Evans and Riley 2023, 63). Healthism also extends beyond the individual, such as when couples work on their health as a joint endeavour, producing a form of ‘relational healthism’ (Robson et al. 2022; also see our discussions of extended healthism in relation to pregnancy and mothering in Evans et al. 2020; Riley et al. 2019). Such expectations for a relational healthism extend to MTAs where many of these technologies offer a ‘partner mode’, such as the spirituality‐themed Stardust app, where the partner mode sends push notifications to a chosen partner, casting spells on them not only to bring things that would comfort the user but also features a ‘sync’ mode where you can add friends who also menstruate into your ‘orbit’ (if those friends accept the invite, i.e., also download the app, the user gets a free month of the premium version, Stardust Super).

Healthism is now a prevalent discourse of health across many countries, especially those structured by neoliberal rationality, including the United Kingdom, the United States of America, Australia, Aotearoa New Zealand, and evident in a range of other European countries including France, Italy, Spain and Finland (see e.g., Cheek 2008; Robson et al. 2022; Turrini 2015). Healthism circulates across multiple actors and agencies, including government health agencies, everyday sense making, and quotidian technologies such as MTAs. Part of this appeal is psychological. As Crawford argued, ‘in a health‐valuing culture, people come to define themselves in part by how well they succeed or fail in adopting healthy practices’ (2006, 403), so that healthism is tied to morality, identity, and notions of living a good life.

The connection to identity makes healthism highly affective. Healthism has an optimistic tone in constructing health as an achievable, stable state, if one works hard enough and consumes appropriately. It also generates a whole scheme of tools and technologies that people can use to create knowledge about their health and assess how well they are is doing (e.g., BMI calculators, calorie counters and health apps). Good feelings are generated when people can fulfil the valued categories, norms and/or subject positions offered.

These optimistic affects are repeated in MTA, where, as the Clue, Flo and Natural Cycles examples above show, empowerment is linked to neoliberal constructs of self‐management, productivity and bodily prediction and control, which are presented as feeling good (also see Della Bianca 2022). Studies show that this language of empowerment (e.g., ‘make empowered health choices’) resonates with MTA users, who report feelings of autonomy and self‐control through better understanding and prediction of their bodily processes (for a review see Riley et al. 2025). However, this incitement to self‐manage can also evoke aversive emotions because framing the subject as solely responsible for their own health elicits anxiety, shame and disgust if their bodies are read as not healthy. For example, MTA users report distress when apps classify their cycles as ‘irregular’, because they interpret their bodies as ‘broken’ even though irregular cycles are typical (Riley and Paskova 2022).

Crawford (1980), and subsequently also Turrini (2015), outlined multiple influences shaping the shift towards healthism, including racism and eugenics, and classed and gendered cultural frameworks. However, gender does not feature heavily in Crawford (1980, 2006) articles on healthism. Gender is absent in the 1980 paper, which focuses instead on class and the elitism of healthism. In Crawford's 2006 paper that updates healthism, gender is minimally considered—discussed in terms of its intersections with class or in a list of multiple elements shaping healthism. For example, women's health movements are listed as one of several anti‐establishment US movements in the 1970s that provided some of the conditions of possibility for healthism. There is also recognition that engagement with fitness might have had a feminist dimension for women, but the potential for healthism to be culturally connected to constructs of female empowerment is not further developed.

A surprisingly small body of literature has since explored the ways in which healthism shapes women's subjectivity and experience. This work covers in a range of contexts including fitness (e.g., Dworkin and Wachs 2009; Markula 1993; Rich 2004; Wright et al. 2006), sexuality (e.g., Tiefer 2000), weight (e.g., Aphramor and Gingras 2009; Harjunen 2021; Tischner and Malson 2012), food and eating differences (e.g., Cairns and Johnston 2015; Nikolova and LaMarre 2023), race (e.g., Thompson 2015), pregnancy (Lind et al. 2023), and technology (e.g., Berry et al. 2021; McMillan 2024). Our contribution to these considerations of gendered healthism is the proposal of a ‘postfeminist healthism’. Below we unpack this concept by first outlining a ‘postfeminist sensibility’, before examining how we understand it in relation to healthism.

### 1.2 Postfeminist Sensibility

Considerations of gender and healthism are limited, despite substantial evidence that women are responsiblised for both their own and other's health, and are often addressed by government, media and commercial organisations in specifically gendered ways in relation to health that align with healthism. Gender and health are thus deeply imbricated, meaning that healthism is inevitably gendered. How healthism is gendered therefore needs to be recognised and theorised. Here, we explain how postfeminist healthism offers an important concept for doing this work.

We draw on Gill (2007, 2017) concept of a postfeminist sensibility, which she understood as a ‘structure of feeling’. This is a term borrowed from Williams (1977) to describe how the sensation of a culture in its contemporary moment emerges through both recognisable patterns of meaning making and also complexities, contradictions, tensions and multiplicity, which unfold into the present in a dynamic process of relations that are highly affective. A structure of feeling allows researchers to recognise that contradictions are part of postfeminism, not different discourses in competition. This is important because, typically, analysts look for coherent and temporarily fixed cultural or psychological patterns. In contrast, in a postfeminist sensibility, disparate and contradictory elements come together and dynamically unfold in response to cultural changes, producing different iterations.

Below, we chart three iterations of postfeminist sensibility that allow us to show changes over time as well as the through‐lines in their logic. This means that iterations do not necessarily supersede the other and that they can exist concurrently with adaptations that respond to cultural, social, and political changes and contexts. Broadly categorised as postfeminism 1.0, postfeminism 2.0, and postfeminism 3.0, we see these less as ‘waves’ of postfeminism, and more as a complex and non‐linear redeployment of similar sets of ideas in emerging contexts, that read together, explain how postfeminism continues to operate as a powerful subjective force.

#### 1.2.1 Postfeminism 1.0

Postfeminist sensibility was first described by Gill (2007) as a form of media address in Anglo‐American media that took concepts from feminism—such as liberation, freedom, and empowerment—but connected these to a consumer‐friendly individualism, often by disavowing feminism (also see McRobbie 2009). This first iteration of postfeminist sensibility was firmly situated as a media representation, located in a recent historical moment before social media and the ‘smart’ mobile phone (and thus apps for digital health and self‐tracking technologies that underpin MTAs). The key elements of this address included:

the notion that femininity is a bodily property; the shift from objectification to subjectification; an emphasis upon self‐surveillance, monitoring and self‐discipline; a focus on individualism, choice and empowerment; the dominance of a makeover paradigm; and a resurgence of ideas about natural sexual difference.

(Gill 2007, 147)

Within this constellation of meaning making, work on the body to meet normative constructs of heterosexual attractiveness, once problematised as objectification, was rebranded as empowerment—representing the ability to transform oneself into an agentic sexy woman, not for a man, but for oneself. Indeed, work on the body's appearance through, for example, make‐up, depilation, exercise and so forth, became a key route through which women could produce themselves as feminine subjects.

Postfeminism 1.0 entangled empowerment with regulation by connecting empowerment to transformation, when transformation required self‐surveillance, examination against norms and work to meet those norms (key tropes of Foucauldian governmentality/modern power). These norms included a media address that made visible a heterosexy, affluent (or economically active and aspirational), classy or middle‐class, youthful, slim, non‐disabled—and often White—femininity (Butler 2013; Dobson 2015; Evans and Riley 2014; Gill 2007; McRobbie 2009). As one of our research participants explained in relation to MTAs, they give off ‘Americanised White women wellness vibes’; whereas Grenfell et al. (2021, 116) describe ‘“typical” users as white, heterosexual, affluent, cisgender women without disabilities’. However, the structural privilege attached these norms is masked through an individualised discourse of choice, reproducing wider patterns of neoliberal rationality.

Making connections between postfeminism and neoliberalism, Gill (2007, 2008) offered postfeminism as a gendered analysis of neoliberalism—an argument that we are echoing here in stating that we need a gendered analysis of healthism. Gill argued that both postfeminism and neoliberalism are structured by an individualism that absents notions of how social or political forces shape lives, and that both produce expectations for a self‐regulating subject—further, that these neoliberal requirements are inherently gendered:

To a much greater extent than men women are required to work on and transform the self, to regulate every aspect of their conduct, and to present all their actions as freely chosen. Could it be that neoliberalism is always already gendered, and that women are constructed as its ideal subjects?

(Gill 2008, 443)

The postfeminist neoliberal subject thus participates in self‐transformation. In postfeminism 1.0 this became a transformation imperative, framing women's bodies as malleable, and thus able to be transformed in order to live a good life. The logic was that since women *could* change themselves, they *should*. Further, transformation towards conventional and normative femininity demonstrated women's empowerment, with the corollary that ‘failed’ bodies—too fat, flabby, unsexy and so forth—evidenced a lack of self‐discipline and self‐regulation required for transformation, and thus moral failure.

Appearance thus became the measure of a woman—connected to neoliberalism and without reference to historical objectification—ushering in a highly judgemental body image culture circulating across a range of women's media, especially makeover shows and magazines that invited viewers to scrutinise and critique (Evans and Riley 2014; Ringrose and Walkerdine 2008). In this scopic regime, a postfeminist gaze developed in the appraising way that women looked at each other and turned that gaze inwards, judging the self (Riley et al. 2016; Rome et al. 2020). Aided by a range of products and services, and promoted by influencers on traditional and social media, postfeminism called forth ‘endless work on the self and that centres notions of empowerment and choice while enrolling women in ever more intense regimes of “the perfect”’ (Gill 2017, 609). The outcome was contradictory expectations of normal‐perfection (Gill 2017, 2023; McRobbie 2020; Riley et al. 2019, 2023).

Another contradictory pairing within postfeminism was in connecting notions of individual choice (e.g., of working on appearance and/or consumption practices) with ideas of natural sexual difference. This (re)turn to biological essentialism means that postfeminist sensibility positions women as free to choose *and* (naturally) choosing traditional feminine pleasures and practices, including shopping, appearance concerns and marriage (Gill 2007; Elias et al. 2016). This element of postfeminism is evident when MTAs employ a biological essentialism that reduces women's diverse social, personal and embodied experiences to their hormones (Ford et al. 2021).

#### 1.2.2 Postfeminism 2.0

We mark postfeminist 2.0 as a response to several cultural shifts including (1) significant public concerns regarding body image problems produced by the appearance‐focused judgemental culture; (2) a rise in popular feminism and digital cultures and (3) entanglements between the transformation imperative and health.

Responding to a backlash against a judgemental body image culture, postfeminist media borrowed from Black, feminist, and fat activism and flipped the narrative, exhorting women to love their bodies (Gill and Elias 2014). However, in the context of a continuing appearance‐oriented culture and expectations of transformation, this means that women are incited to manage the contradictory demand of loving their existing body and wanting to transform it. The ‘love your body’ discourse thus becomes stripped of its politics while intensifying the transformation imperative because it prescribes women to love their body and undergo psychological transformation to do so.

Rather than providing an alternative to postfeminism, the turn to positivity was incorporated into postfeminism, extending its transformation imperative to include practices of *mental* good health, such as body positivity, self‐love, self‐care, and confidence (Calder‐Dawe et al. 2021; Gill 2023; Evans and Riley 2023; Riley et al. 2023). Folded into the logic of postfeminism, self‐care is both empowering and regulatory, understood as a valued practice of self‐enhancement, yet requiring constant self‐management that consumes a range of resources including time, money and energy that make (potentially excessive) demands of women. In the extract below, for example, one of our MTA research participants oscillated between constructing tracking as a form of self‐care or of self‐regulation, an unresolvable tension within postfeminism:

self‐care is just one more thing that women have to do, like one more thing on the list that we have to do for ourselves [group laughter] … I don't want to do my self‐care anymore. Somebody else can do my self‐care [laughing] … [But] this is what I do to look after myself. This is part of valuing my my own body and my own system, I suppose.

The borrowing of feminist body positivity is part of a wider shift towards feminism that characterises postfeminism 2.0. This included a rising popular feminism, responding, in part, to #MeToo and the first election of Donald Trump in the USA and his infamous ‘grab them by the pussy’ comment, which were indicative women's lack of gender equity—and fuelled by the affordances of social media, which allowed online campaigns as well as on/off‐line crossovers (Banet‐Weiser 2018). For some, this signalled a shift away from postfeminism. But for others, it demonstrated the adaptability and flexibility of a postfeminist sensibility because responses to these issues—although no longer repudiating feminism—had a distinctly postfeminist flavour. For example, forms of self‐management, responsibilisation, and individualism co‐existed with popular feminism, as in the feminist subject who, in ‘leaning in’ (Sandberg 2013), did not fight the system but fitted into corporate business and liberated themselves through capitalist structures (Rottenberg 2018).

Such adaptations demonstrate another similarity between postfeminist sensibility and neoliberalism in its mobility in, and through, different local contexts (Ong 2006); indeed, many scholars have traced postfeminism as accompanying neoliberalism in (once) emerging markets such as India, Russia and China, forming ever more intricate entanglements with global flows of neoliberal capitalism and associated forms of citizenship. For example, Zhang and Riley (2024) analysed the synergies between postfeminism and Chinese government ‘positive energy’ policies in Chinese reality television (see also, Yang 2020).

Despite popular feminist resurgence and body acceptance, postfeminist tropes of empowerment through bodywork were not abandoned, but instead, reinvigorated through connections to health. Reproducing healthism, health became presented as an ideal, moral state produced through work on the body and self that, by intersecting with postfeminism, could be read through appearance—such as having a slim body in the context of a weight‐framing of health. For example, motivational fitness content on social media (dubbed ‘fitspo’) continued to represent the thin ideal through associations with health that oriented to (popular) feminist discourses of women's power, such as in the aphorism ‘strong as the new skinny’ (Riley and Evans 2018; Evans and Riley 2023).

The conflation of health and appearance occurs at multiple levels, including in healthcare settings when, for example, dentists advertise lip fillers (Riley et al. 2023); the rebranding of restrictive eating as not dieting, but healthy (Cairns and Johnston 2015); and how a range of appearance‐related consumer practices were connected to health, including, for example, vaginal steaming (Vanderburg and Braun 2017). New experts of health and fitness became a whole influencer industry, coming to play a significant pedagogic role for understanding health (Camacho‐Miñano and Gray 2021), or, in the context of MTA, reproductive health (Geampana 2024). Entanglements between health, beauty and wellness are also evident in MTA. For example, Friedlander's (2023, 694) research with young people shows how health and appearance concerns are felt with a ‘strongly affective dimension’ through the app, especially in the way the app conflates fatness/slimness with unhealthy/healthy.

This shift towards health as a marker of success often aligns with White, middle‐class or affluent norms, masking structural inequalities related to the financial barriers to participation in not only consumer‐driven health cultures but also racialised, classed, fat‐phobic, and disabled marginalisation. Promoted largely on Instagram, clean eating, for example, celebrated thin, young middle‐class White women who valorised expensive ingredients and appliances as embodiments of wellness, leaving many women judging themselves against these images of apparently easily lived normal‐perfection and feeling a lack (Cairns and Johnston 2015; O’Neill 2020; Riley et al. 2023; Wilkes 2020).

The turn to health also enables a revitalisation of judgement of others. Although no longer acceptable to judge someone for their appearance, the interconnections of postfeminism 2.0 and healthism meant people could judge on the basis of health since the logic of healthism gave appearing healthy a moral framing. Hateful comments responding to images of plus‐sized model Tessa Haliday exercising, for example, were framed through discourses of care and concern for her health.^4^

This intersecting constellation of postfeminism and healthism further intensifies affective registers, not only circulating hope, optimism and pride, but also anxiety, shame and fear (Evans and Riley 2023; Riley et al. 2023, 2016). Hope and optimism attach to the idea that people might achieve this valued state of health‐appearance‐lifestyle, and those who achieve it often experience pride and pleasure, both through their own assessments and cultural validation. But given how this construct of health is often unachievable or unsustainable, the connection of postfeminism to health also circulates anxiety, fear, shame, jealousy and even hatred.

The intensity of these feelings is evident in how all bodies are illuminated, so that even in the context of thin privilege, thin people experience ‘skinny shaming’ and over attention to their bodies, including jealousy. Perhaps counterintuitively, fat people can also experience jealousy, as one of the many forms of micro and macro aggressions they experience for ‘failing’ to meet ideal health‐femininity‐appearance norms (the jealously relating to an often false perception that they are free from oppressive cares of appearance/health concerns). The violence of racism also amplifies this stigma. For example, fat Black women report feeling excluded or problematised, even in ‘inclusive’ gyms (Bailey et al. 2023).

#### 1.2.3 Postfeminism 3.0

Post‐COVID, we chart a deepened sense of anxiety and precarity. The experience of COVID‐19 not only heightened health concerns for many but also enabled digital cultures of disinformation and misinformation. These digital cultures largely emerged from a wellness industry already in tune with postfeminist sensibilities that locate health concerns as an individual responsibility (Baker 2022). Outside of conspiracy theory, we also track a continuing intensification of the transformation imperative, with influencers circulating expectations to work on health, appearance, mind, relationships, sex and career, blurring the boundaries between these spheres and intensifying expectations to both work on, and succeeded in, living a perfect life (Kurghan and Thorpe 2025; Peticca‐Harris et al. 2024; Vanderburg and Braun 2017). The outcome is an affectively charged understanding that an optimised life is both exceptional and attainable, and the disciplinary work towards it is empowering.

The celebratory tone of popular feminism has also receded in the context of a culture war on gender and the emergence of reactionary figures and feminisms, such as Andrew Tate, ‘trad wives’, and far‐right politics (Kay 2024). Sykes (2024), for example, showed how women content creators on TikTok make highly stylised performances of resource intensive domestic work framed within a postfeminist sensibility as normal, natural and fulfiling. The potential for postfeminist tropes to merge with reactionary politics is evident and may form defining elements of postfeminism 3.0. In relation to MTAs, for example, in the context of a new set of geopolitical control over bodies and health related to the overturning of Roe versus Wade and re‐election of Trump, anti‐abortion supporters such as Peter Thiel have financed menstrual tracking apps that promise to allow women to ‘become the expert of you’ and ‘retake control of your body’.^5^ Concurrently, the problematic promotion of Ozempic as a weight control intervention heralds a return to a thin ideal, based on the flawed logic underpinning both healthism and postfeminism that people now have a choice, and therefore should not choose to stay fat. In her analysis of happiness, Ahmed (2010) analysed how constructs of happiness can be used to direct people's desires towards the status quo—in postfeminism 3.0 we track an unhappy shift of using the language of choice, happiness and pleasure to go beyond supporting the status quo and towards reactionary constructs of gender.

## 2 Postfeminist Healthism

The iterations of postfeminist sensibility outlined above are part of a dynamically shifting structure of feeling, that, although composed of multiple and contradictory elements, is aligned with healthism. Despite this alignment, and that both have significant leverage within the Academy—3779 citations for Gill's (2007) original formation; and 3072 for Crawford's (1980)—only a small body of research interrogates the relationships between these concepts. Noticeable exceptions include Dubriwny (2012), Cairns and Johnston (2015), and Rich (2018), but even here, the synergistic relations between postfeminism and healthism are not significantly expounded.

Addressing this gap, we developed the concept of ‘postfeminist healthism’ (Riley et al. 2019; Evans et al. 2020). Postfeminist healthism describes a cultural imperative for women to work on their body and mind, in order to meet narrow cultural ideals of healthy embodied femininity, and to understand this work as empowering and a measure of their morality and value, with any failure to do so located within the individual. Folded into this understanding of postfeminist healthism is a transformation imperative that connects contemporary individualised and responsibilised notions of health in healthism to (historical) constructions of femininity as always‐already flawed but fixable if, with a postfeminist twist, people work on themselves.

Given the extensions of health we discussed earlier, this ‘health’ work occurs across multiple sites, creating a myriad of health, including lifestyle, relationships, work–life balance, ‘sleep hygiene’, and so forth, with often impossibly high expectations of what good health looks like in each. What emerges then, is a ‘normative expectation for women to be confident, sexually agentic, efficacious and successful in their life plans for public roles, paid employment, intimate relationships and embodied health’ (Riley et al. 2019, 6). In a context where ‘good’ health is positioned as both exceptional and normal, and with multiple ideas standing in for ‘good’ health, it becomes hard to actually know what good health should be, creating uncertainty when navigating health.

Moreover, achieving perfection means meeting multiple demands on health that can themselves be contradictory (e.g., working on relationship health may mean not working on body health). Thus, even making apparently ‘good health’ choices ‘comes with a high amount of risk, control, and anxiety, that actually disempower people … through practices that might make feeling healthy and happy less likely to happen’ (Riley et al. 2023, 162). The outcome is an anxious and precarious concept of health that requires both a reliance on expert knowledge, self‐education and the development of complex ‘lay epistemologies’ through which people come to understand and manage their health (Crawford 2006; Robson et al. 2024).

In previous work, we have shown the utility of applying postfeminist healthism to a range of gendered health issues, including self‐help, weight, cosmetic surgery, eating, exercise, pregnancy, sex, caregiving and body image (Evans et al. 2020; Riley et al. 2019, 2023). Although varying in their engagement with it, others have applied the concept of postfeminist healthism to explore constructions of health in women's health magazines (Beijbom et al. 2023), aesthetic labour (Prohaska 2023; Prohaska and Thompson 2024), fitness and health education (Kurghan and Thorpe 2025; Rich et al. 2020), menopause (Jermyn 2023), food and eating (Lewthwaite and LaMarre 2022), pregnancy (Fassbender 2021), and alcohol consumption (Kersey et al. 2025).

In relation to MTAs, Riley and Paskova (2022) offer another example of applying a postfeminist healthism lens, which they used as part of their theoretical framework considering how the social context, affordances of digital technology, and bodily processes shape the conscious experience of menstruation. Developing this work, here we highlight how the matrix of postfeminist healthism empowerment discourses intersect with neoliberalism and consumerism. For example, in the context of many sanitary product companies developing their own MTAs, the webpage for Bodyform's MTA suggests that, ‘Your period should never stop you living your life, but if you're worried about that big event or holiday then our tracker can help you plan ahead’,^6^ with a link to products needed when menstruating, and including the advice to always have a clean pair of ‘period pants in your bag or car’. Thus, menstruation is understood as an attitude to a particular lifestyle, an opportunity to consume and requiring careful planning.

As Pichon et al. (2022, 394) stated, ‘Menstruation is an embodied, leaky experience, but apps flatten the experience and compel menstruators to hide and control their menses’. Alongside flattening the experience, we would argue that a postfeminist healthism means that such control is accepted as part of the expectation to be a productive citizen, engaging in the economy for work and leisure, and organising menstruation around ones' career and lifestyle choices. Postfeminist healthism thus presents itself as a healthy way of living—even as it might limit human flourishing or broader notions of empowerment.

### 2.1 The Subjectifying Power of Postfeminist Healthism

Postfeminist healthism is reproduced in MTAs, but that does not explain why so many people download and use them. After all, the forms of self‐discipline, self‐objectification and incompatibility with actual lived bodies that can produce a cruel optimism and negative affects might make MTAs an unpopular technology. In this final section, we therefore map out key elements that we see as enabling postfeminist healthism to be an intelligible force that acts so powerfully on subjectivity—hooking onto and shaping people's desires and sense of self. These elements focus on (1) how a postfeminist healthism is highly affective and operates through norms and socially valued identities that become part of, not separate to, people's inner thoughts and feelings and (2) how resistance is recuperated in ways that map onto the practices of non‐linear warfare.

#### 2.1.1 Affect

We theorise postfeminist healthism as an affective force working through both desires to mitigate fear, anxiety and shame on the one hand, and on the other, to experience the pleasures of self‐mastery and self‐worth that comes from recognition and validation. As Crawford's (1980) discussion of healthism demonstrates, what is understood as good health is not just desirable, but a mark of a good life and connected to happiness (as in the normative coupling of ‘health’ and ‘happiness’ (Riley et al. 2019)). Affect in this sense is ‘sticky’, attaching itself to certain objects including health (Ahmed 2010).^7^

Health, however, is a fundamentally precarious notion to attach to happiness, especially when understood as an individual responsibility, since often ill health is not within our control. Further, a postfeminist healthism that shapes understandings of good health often mean it is multiple (body, mind, relationships, career); exceptional (aligning with the perfect) and extended (such as when otherwise typical bodily functions fall into something to treat as problematic and in need of risk management, as when MTA classify users' cycles as ir/regular because it is easier to predict, but is interpreted by users with irregular cycles as a problem with their bodies). In making sustained health unachievable for many, postfeminist healthism thus circulates anxiety, fear and shame, both for those who never achieve this limited construct of health, and for those who do but feared losing it (Riley et al. 2023; Evans and Riley 2023; Riley et al. 2025).

MTAs thus promise control of the uncontrollable. Hope and optimism are attached to the idea that people might achieve this valued state of a healthy lifestyle; whereas pride, pleasure and cultural and personal validation circulate when it is achieved. Good health in the context of MTAs therefore make sense in relation to Berlant (2011) concept of cruel optimism. Questioning why we remain attached to capitalism when it generates unequitable social structures that, for the majority, exhaust and wear people out, Berlant proposes that this power comes from the promise that the good life is achievable. Berlant (2011, 1) identifies the ‘simplest’ cruel optimism as being ‘a new habit that promises to induce in you an improved way of being’. The habit of entering, to various degrees for different MTAs, symptoms, moods, libido, sexual activity, vaginal discharge, temperature, breast tenderness, menstrual flow and so forth is thus one that promises happiness. Relatedly, our research participants talked about such data entry practices produced a sense of safety and associated comfort—that, in the context of struggling with their reproductive health, epistemic injustice (a typical finding in women's health research), and poor healthcare gave them a sense of agency—at least they were doing something. In this context, MTA users attach their hope onto the app for an improved way of being, and even if that improvement does not materialise, the *performance* of good health counted for something—it could give them hope.

#### 2.1.2 Norms

Norms render people ‘knowable’ in particular ways and allow them to be evaluated and then managed in relation to how much they conform or deviate against these standards (Foucault 1980; Foucault 1988). Where postfeminist analysis has identified this self‐surveillance in seeing others, judging, and internalising this judgemental gaze onto the self (Gill 2023; Riley et al. 2016; Rome et al. 2020), MTAs make the self the object of comparison, evaluated by the app and based inputted data—producing the norms‐surveillance‐examination pattern of how modern power works on and through psychology as one learns to understand oneself through these forms of knowing.

Thus, norms become part of people's thoughts and feelings, not separate to them, meaning that they are part of a person's thoughts and feelings, which means risking pain and discomfort to reject them because we have an attachment to them (Davies 2013). This attachment can also be projected out to others, producing significant forms of social policing for those who do not meet norms. Connecting back to our discussion of affect, there is pleasure in social validation—of being understood by oneself and others as a good person that allows postfeminist health to hook onto our desires—but it is also met by an equal and opposite response of fear, of both self and other, hatred, aggression, policing and so forth. The ‘doing it for myself’ account of postfeminist empowerment thus masks significant socially produced external and internalised pressure to participate in postfeminist healthism and produce oneself into these valued norms. A pressure unlikely be legible for many people schooled in understanding themselves only through neoliberal individualisation that excludes other forms of knowing.

#### 2.1.3 Non‐Linear Warfare

Affect and norms may hold people's attachments to postfeminist healthism, but gaps between the promised good life and reality open possibilities to challenge the logic of postfeminist healthism. However, two powerful elements can maintain postfeminist healthism: individualism and recuperation.

At a personal level, the individualism underpinning postfeminist healthism means any ‘failure’ can be located in the individual—such as a personal failure to log the right, or enough, data—an interpretation made more likely in the in the context of complex and contradictory constructs of health. The hegemony of this thinking, tied in as it is to neoliberalism, also means that there are few easily accessible alternative frameworks to draw on, especially as what appear to be alternatives may turn out to be just other versions of postfeminist healthism, such as how self‐care becomes another thing that women need to work on.

Sometimes the gap between the desired good life and reality is too big to ignore at a social level. However, even in this context, critique, and associated attempts to do things differently, gets recuperated. We gave an example in our discussion of postfeminism above, in how the critique of body image judgemental culture that was part of postfeminism 1.0 was maintained into postfeminism 2.0 through appropriation of body positivity. Central to our understanding how postfeminism continues in the face of critique, is a concept we borrowed from political science, of non‐linear warfare.

Non‐linear warfare encompasses new forms of political practices of implementing contradiction, confusion, and critique in the political sphere which generates a desire for safety, security, and normativity (Riley et al. 2023; Curtis 2014; Pomerantsev 2014). The multiple contradictions of postfeminism (e.g., to work on one's body and unconditionally love one's body; to understand that a healthy body is accessible to all and requires significant resources of time, money, classed and racialised privilege) creates confusion and a desire for the safety of normativity, folding people back into working on themselves. Health understood through nonlinear warfare shows how these contradictions hold the system together, rather than providing a route to challenge it.

Similarly, where once critique might have been able to challenge such a system, within non‐linear warfare, critique is absorbed into the system allowing it to continue. For example, postfeminism adapted to critiques of its body judgemental culture by reframing dieting practices as forms of joyful, healthy eating; or when well‐being influencers were outed for promoting unhealthy practices, they confessed the mental and physical damage they too experienced from attempting to live a ‘healthy lifestyle’ only to promote a new one that addressed these problems. An important lesson for critical scholars then, is that doing critique is not enough.

#### 2.1.4 There Is a Crack in Everything. That's How the Light Gets in

Despite how postfeminist health folds individual and social resistance back into itself, there are also examples of cracks in the system. Martinussen et al. (2020), for example, identify moments in women's friendships that provide ease, escape and refuge from postfeminist imperatives for self‐improvement and self‐surveillance. In relation to MTAs, Riley and Paskova (2022), highlight a ‘pedagogy of appreciation’ in which women with premenstrual syndrome (characterised by pain and intense moods that are both unpleasant and stigmatised) learnt to understand these embodied experiences in an affirmative way through their app use. MTAs allowed them to understand the menstruating body through pride and awe, opening ‘capacities to differently know and love the existing body (bleeding, painful and awe‐inspiring), rather than work on it with the hope of fixing it or making it loveable’ (11). While Algera (2023, 256) offers another crack, in how the continuous dynamic between MTA notifications representing users' cycles and MTA user's experience of their bodies sensations cultivated awareness of their lived physiology, so that app use enabled users to develop ‘an embodied *skill*: to *feel* the body's reproductive physiology’.

These examples offer moments of escape from postfeminist healthism, since friendships can also reinforce postfeminist transformation imperative; MTAs reproduce limited postfeminist notions of empowerment as work on the self; and celebrating an embodied skill may turn it into an imperative. For both individuals and scholars of healthism, the lessons offered in these examples appear to be of grabbing those small moments and using them to widen the cracks while always recognising that the system will seek to recuperate itself.

## 3 Conclusion

This article advances the concept of postfeminist healthism to address a critical gap in theorising the gendered dimensions of contemporary health discourses. We have demonstrated that healthism is always already gendered, and that postfeminist healthism operates as a powerful subjective force—one that persists despite critique or evident harm. Through this lens, we have analysed how health becomes imbued with affective promises of empowerment, certainty and moral worth while simultaneously demanding self‐surveillance, discipline and alignment with normative gendered expectations. These dynamics are sustained through affective attachments to health ideals and the recursive incorporation of critique into further self‐optimisation or structural adaptation, akin to non‐linear warfare.

Our use of MTAs as a case study illustrates the utility of postfeminist healthism as a framework in unpacking the entanglements of pleasure and anxiety, and, empowerment and regulation, within digital health technologies. Despite limited engagement with postfeminism in MTA research, we show how this conceptual framework illuminates the complex ways users negotiate health, embodiment and identity. Overall, we have shown how postfeminist healthism acts as a powerful subjective force, even in the face of critique or recognisable harm, and invite researchers to engage with it in their studies of healthism.

## Author Contributions

**Sarah Riley:** conceptualization, formal analysis, writing – original draft, writing – review and editing. **Adrienne Evans:** conceptualization, formal analysis, writing – original draft, writing – review and editing. **Martine Robson:** conceptualization, formal analysis, writing – original draft, writing – review and editing.

## Data Availability

Research data are not shared.

## Endnotes

^1^
The research data comes from cooperative inquiry group discussions with people living in Aotearoa New Zealand using MTA, funded by the Royal Society Te Apārangi, grant number MFP‐MAU2210.

^2^
For example, the NHS recommends tracking menstruation for a range of health issues connected to menstruation, fertility, and endocrinological and hormonal functions.

^3^
Flo offers a range of ‘commitments’ depending on the users' inputted data—this one is generated for the user looking to track perimenopause.

^4^
While framed within discourses of care they were fat phobic.

^5^

https://28.co.

^6^

https://www.bodyform.co.uk/period‐tracker/#.

^7^
We also follow Ahmed (2010) in seeing affect and emotion as related, both discursive and pre‐discursive, social and psychic—see Evans and Riley (2023) for a full discussion of how we understand and apply affect theory.
